# CAMP: A Context-Aware, Multimodal, and Privacy-Preserving Pedestrian Trajectory Prediction Framework

**DOI:** 10.3390/jimaging12050197

**Published:** 2026-05-02

**Authors:** Bin Yue, Shuyu Li, Anyu Liu

**Affiliations:** School of Artificial Intelligence and Computer Science, Shaanxi Normal University, Xi’an 710119, China; yuebin@snnu.edu.cn (B.Y.); lay926@snnu.edu.cn (A.L.)

**Keywords:** trajectory prediction, multimodal fusion, potential field, optical flow, differential privacy

## Abstract

Pedestrian trajectory prediction is vital for crowd analysis and human–-robot interaction. Recent deep models enhance accuracy by modeling social interactions and scene context, but they often remain opaque and rarely address privacy risks associated with learning individualized motion patterns. We propose CAMP, a Context-Aware, Multimodal, and Privacy-preserving pedestrian trajectory prediction framework designed around a role-aligned multimodal architecture, in which trajectory representations, dynamic scene cues, and explicit spatial interaction constraints are modeled through complementary branches. In CAMP, the trajectory encoder separates shared motion regularities from individualized motion tendencies, the optical-flow encoder captures motion-centric transient scene dynamics, and the potential-field encoder provides an interpretable spatial cost prior for obstacle avoidance and social interaction modeling. A Transformer-based decoder fuses these modalities to predict future trajectory distributions. To reduce the exposure of personalized motion patterns, we apply targeted DP-SGD only to the individual branch during the private fine-tuning stage, while treating the remaining frozen components as post-processing under the stated threat model. Experiments on the ETH/UCY benchmark show that CAMP achieves competitive ADE/FDE performance under the reported setting, while its private variant DP-CAMP maintains a reasonable utility–privacy trade-off across several reported privacy budgets.

## 1. Introduction

Forecasting pedestrian trajectories is a fundamental problem in intelligent transportation, autonomous driving, and human–robot interaction. Accurate prediction of future pedestrian motion is important not only for safe and smooth vehicle–pedestrian interaction, but also for the practical acceptance of autonomous systems in real-world environments. However, reliable trajectory prediction remains challenging in complex urban scenes, where pedestrian motion is jointly influenced by individual intent, social interaction, and surrounding environmental structure [1].

Deep learning has driven significant progress in trajectory prediction. Recurrent models such as RNNs/LSTMs [2,3], graph neural networks (GNNs) [4], and attention-based architectures [5,6] have significantly improved predictive performance by learning spatio-temporal dependencies from data. Nevertheless, most data-driven models still suffer from two limitations. First, their prediction process is often difficult to interpret, which weakens their transparency in safety-sensitive applications. Second, methods relying mainly on historical coordinates may fail to capture rich contextual cues from the surrounding scene. Prior studies have shown that multimodal inputs, such as semantic maps, optical flow, and human pose, can provide complementary information and improve robustness [7,8]. However, many existing approaches employ relatively shallow fusion strategies, making it difficult to fully exploit fine-grained dependencies across modalities. These limitations motivate the development of a framework that can better integrate motion history, scene dynamics, and interaction constraints. As trajectory prediction moves closer to practical deployment, privacy concerns also become increasingly important [9,10]. Pedestrian trajectories may reveal individualized behavioral patterns, and models trained on such data may inadvertently encode sensitive motion tendencies. Although recent methods have achieved strong predictive accuracy, most do not explicitly discuss how privacy risks associated with individual-specific motion patterns are handled during model training and adaptation. This gap is particularly important when personalized information is introduced into the learning process. Therefore, beyond improving prediction accuracy, it is also necessary to consider how to incorporate privacy-aware learning mechanisms into multimodal trajectory forecasting. To address these issues, we introduce CAMP, a Context-Aware, Multimodal, and Privacy-preserving pedestrian trajectory prediction framework. CAMP is designed to jointly consider three aspects of the task: predictive accuracy, interpretability, and privacy protection. CAMP further adopts a targeted privacy strategy, where DP-SGD is applied only to the individual branch during private fine-tuning under the stated threat model. In this way, CAMP aims to protect personalized motion patterns during private adaptation while preserving the utility of shared motion and scene representations learned from public data.

Our main contributions are summarized as follows:

A role-aligned multimodal trajectory prediction framework. Rather than simply combining multiple context sources, CAMP is designed around a structured division of modeling roles across representations and modalities. The trajectory encoder captures motion intent while explicitly separating shared motion regularities from individualized motion tendencies. The optical-flow branch models motion-centric transient scene dynamics, and the potential-field branch provides an explicit spatial cost prior for interaction-aware forecasting. This design allows different sources of predictive information to contribute in a complementary rather than redundant manner.

A unified integration of dynamic context, explicit interaction constraints, and privacy-aware adaptation. CAMP distinguishes between two types of contextual information that are often treated in a homogeneous way in existing multimodal predictors: transient scene motion cues and explicit spatial interaction constraints. We encode them through separate optical-flow and potential-field branches and fuse them with trajectory features through layered cross-attention. Based on the representation split in the trajectory encoder, we further couple private adaptation with the individualized branch only, so that the location of the privacy mechanism is consistent with the part of the model intended to capture personalized motion tendencies.

A targeted differential privacy strategy under a clearly stated fine-tuning scope. Instead of applying privacy perturbation uniformly to the entire model, CAMP applies DP-SGD only to the individual branch during private fine-tuning, while keeping the generic branch and the context encoders frozen. Under the stated threat model, this design reduces the dependence of the adapted model on private individualized motion patterns while retaining shared motion and scene knowledge learned during public pretraining.

Extensive experiments on the ETH/UCY benchmark. The experimental results show that CAMP achieves competitive ADE/FDE performance under the reported setting, while its private variant maintains a reasonable utility–privacy trade-off across several reported privacy budgets. The ablation and efficiency analyses further indicate that the gains of CAMP come from the complementary roles of its modality branches rather than from a simple accumulation of inputs.

The rest of the paper is organized as follows. Section 2 reviews related work. Section 3 introduces the preliminaries of differential privacy and the threat model considered in this work. Section 4 details the proposed CAMP framework. Section 5 describes the experimental setup and analyzes the results. Finally, Section 6 concludes the paper and discusses future directions.

## 2. Related Work

Accurate pedestrian trajectory prediction requires effective modeling of human–human and human–environment interactions. Early studies mainly used interpretable model-driven formulations such as the social force model (SFM) [11], which characterizes motion by goal attraction, social repulsion, and environmental constraints. Subsequent work extended these formulations by incorporating learnable physical parameters or combining motion dynamics with contextual information [12,13]. Although these approaches provide intuitive behavioral explanations, they usually rely on hand-crafted assumptions and often generalize poorly in complex real-world scenes.

With the development of deep learning, research has gradually shifted from explicit rule-based modeling to data-driven interaction modeling. Early recurrent approaches, such as Social-LSTM [2], introduced social pooling to encode neighborhood interactions, while generative methods such as Social-GAN [3] further modeled multimodal futures. Later studies improved diversity, stability, and social consistency by introducing additional priors or constraints, including energy-based formulations [14]. To capture longer-range and more complex spatio-temporal dependencies, graph neural networks (GNNs) and Transformers have become widely adopted. GNN-based models such as Social-STGCNN [4] and STGAT [15] represent pedestrians as nodes in a spatio-temporal graph and learn interactions through message passing or attention. Recent advances optimize graph structure and attention patterns using deformable spatio-temporal convolutions [16], sparse directed attention [17], or unified spatio-temporal graphs [18]. Subsequent researchers have developed various improved models based on the Transformer. STAR [5] jointly models temporal dynamics and interactions among multiple agents through spatio-temporal attention, and AgentFormer [6] creates a combined temporal and agent attention space to explicitly model the coordination among multiple agents over the long term. Building on these foundations, variants like STGSTN [19] introduce sparse spatio-temporal graphs for efficiency, while STGlow [20] uses a generative flow-based framework to learn complex trajectory distributions and generate realistic, diverse forecasts. In addition, recent stochastic trajectory predictors, such as sampling- and diffusion-based methods, have demonstrated strong performance in modeling multimodal futures, highlighting the increasing importance of expressive generation mechanisms in this field [21,22,23,24].

Relying only on historical coordinates is often insufficient for representing scene context and transient motion cues, which motivates multimodal trajectory prediction. A common direction introduces static scene information, such as bird’s-eye-view images or semantic maps, to provide priors on roads, sidewalks, and obstacles. For example, GST-MR [25] models human–environment interaction using scene segmentation within a graph-based spatial Transformer, while SIF-TF [26] strengthens scene semantics with a dedicated scene-interaction fusion Transformer that grids and pools semantic features to fuse scene and social cues. Some methods use optical flow to capture global motion patterns and transient dynamics. MTN [27], for instance, extracts optical flow from both global scene regions and pedestrian-related regions, and performs multi-level fusion to capture transient motion relationships. Human pose has also been used as an auxiliary modality to improve intent inference. DDGCN [8] learns dynamic spatiotemporal correlations from skeletal sequences to enhance motion understanding further. These studies show that multimodal inputs can improve predictive performance by providing complementary scene and motion cues. However, many existing methods mainly focus on accuracy gains and provide limited discussion of interpretability.

As trajectory prediction moves toward practical deployment, privacy protection has become an increasingly important consideration. Existing efforts can be roughly grouped into three directions. The first direction reduces direct exposure to sensitive visual appearance by replacing raw RGB inputs with alternative sensing or representation forms, such as LiDAR, skeletons, or motion-based cues [28,29,30]. These approaches may reduce direct dependence on facial or appearance information. The second direction relies on decentralized training, such as federated learning, to keep data on local devices, while personalized federated variants further address data heterogeneity [31,32,33]. The third direction introduces formal privacy guarantees through differential privacy (DP) [34], typically implemented via DP-SGD. However, directly perturbing all latent representations or relying only on output perturbation [9,35] often leads to noticeable utility degradation. Therefore, a central challenge is how to preserve individualized motion privacy while retaining shared spatio-temporal and scene-level knowledge.

Existing studies have advanced interaction modeling, multimodal context fusion, and privacy-preserving learning from different perspectives, but these directions are still rarely connected through a unified design principle. In particular, many existing methods do not explicitly distinguish what should be modeled by trajectory representations, what should be contributed by dynamic scene cues, and where privacy constraints should be imposed during adaptation. CAMP is designed to address this gap through a role-aligned architecture. The trajectory encoder separates shared motion patterns from individualized motion tendencies, the optical-flow branch captures motion-centric transient context, and the potential-field branch provides an explicit and interpretable spatial cost prior for socially compliant forecasting. Based on this decomposition, private adaptation is restricted to the individualized branch, making the location of the differential privacy mechanism consistent with the part of the representation intended to encode personalized motion information. In this sense, the contribution of CAMP is not merely the aggregation of several existing modules, but the construction of a unified framework that aligns representation decomposition, multimodal context modeling, and privacy-aware adaptation within a single trajectory prediction pipeline.

## 3. Preliminaries

In this section, we briefly review the notions of differential privacy (DP) used in this work and clarify the threat model under which the privacy guarantee of CAMP is discussed.

### 3.1. Differential Privacy

**Definition** **1.** $\left(\varepsilon ,\delta \right)$ *-differential privacy [34]*. *Let* A *be a randomized algorithm that takes a dataset as input and outputs a random variable in some space* O *. We say that* A *satisfies* $\left(\varepsilon ,\delta \right)$ *-differential privacy if, for all neighboring datasets,* $D,D^{\prime }$ *, and all measurable subsets,* S⊆O*:*

(1) $$Pr\left[A\left(D\right)\in S\right]\leq e^{\varepsilon }Pr\left[A\left(D^{\prime }\right)\in S\right]+\delta$$

*where  *ε>0 *is the privacy budget and* δ≥0 *is a small failure probability. Smaller* ε *and* δ *correspond to stronger privacy protection.*

**Theorem** **1.** *Post-processing [34]*. *Let* A *be an* $\left(\varepsilon ,\delta \right)$ *-DP algorithm and let* f *be any randomized mapping that does not access the original dataset. Then, the composed algorithm* $D\to f\left(A(D)\right)$ *is also* $\left(\varepsilon ,\delta \right)$ *-differentially private.*

In CAMP, this property ensures that once individual-level features have been learned using a DP mechanism, any subsequent processing that only accesses those features preserves the same privacy guarantees.

**Theorem** **2.** *Differentially private stochastic gradient descent (DP-SGD) [36]*. *Let* θ *denote the parameters to be trained under DP, and let* $l\left(\theta ;x\right)$ *be the per-example loss for a data point* x *. At each iteration, DP-SGD samples a minibatch* B *from the dataset and proceeds as follows:*

*Step 1: Per-example gradients. Compute the gradient for each example* x∈B:

(2) $$g_{x}=\nabla_{\theta }l\left(\theta ;x\right).$$

*Step 2: Gradient clipping. Clip each gradient to a fixed* $l_{2}$ *-norm bound* C>0:

(3) $${\tilde{g}}_{x}=g_{x}\cdot min\left(1,\frac{C}{∥g_{x}{∥}_{2}}\right).$$

*Step 3: Noise injection and aggregation. Aggregate the clipped gradients and add Gaussian noise:*

(4) $$\bar{g}=\frac{1}{\left|B\right|}\sum_{x\in B}{clip}_{C}(g_{x})+N(0,\sigma^{2}C^{2}I),$$

*where* σ>0 *is the noise multiplier.*

*Step 4: Parameter update. Update* θ *using* $\bar{g}$ *in the same way as in standard SGD or Adam.*

The privacy loss incurred by repeatedly applying this mechanism can be bounded using Rényi differential privacy (RDP) accounting for the subsampled Gaussian mechanism, and then converted into an overall (ε, δ) guarantee.

### 3.2. Threat Model and Scope of Privacy Guarantee

In this work, the formal privacy guarantee of CAMP is discussed with respect to the private fine-tuning stage of the individual branch. We assume that the model is first pretrained on public data to learn shared motion and scene representations, and is then adapted on private trajectory data by updating only the individual branch with DP-SGD. Accordingly, the protected data in our analysis are the private trajectories used during this fine-tuning stage.

We consider a black-box adversary who can query the released model with trajectory inputs and observe its prediction outputs, but does not directly access the private fine-tuning trajectories. Under this threat model, the privacy concern is whether individual-specific motion patterns from the private fine-tuning data may be memorized and exposed through the adapted model. Therefore, the formal DP guarantee in CAMP is defined with respect to the dependence of the individual branch on the private fine-tuning dataset.

It is important to clarify the scope of this guarantee. Freezing the generic branch and the remaining modules during private fine-tuning prevents them from absorbing additional information from the private adaptation data, but it does not retroactively remove information that may have been encoded during pretraining. Therefore, the formal privacy guarantee discussed in this work does not cover the entire pretraining pipeline. Instead, it applies to the private fine-tuning process of the individual branch under the threat model described above.

## 4. Proposed CAMP Framework

This section introduces the proposed CAMP framework, which aims to balance accuracy, interpretability, and privacy in pedestrian trajectory prediction. Its main goal is to generate forecasts by explicitly modeling and combining three essential information streams: (i) trajectory-based motion intent, encoded by the trajectory encoder; (ii) dynamic scene motion cues, represented by the optical-flow encoder; and (iii) interaction constraints, modeled by the potential-field encoder. These features are subsequently fused by a multimodal decoder to generate future trajectory distributions.

### 4.1. Overall Architecture

**Figure 1:**
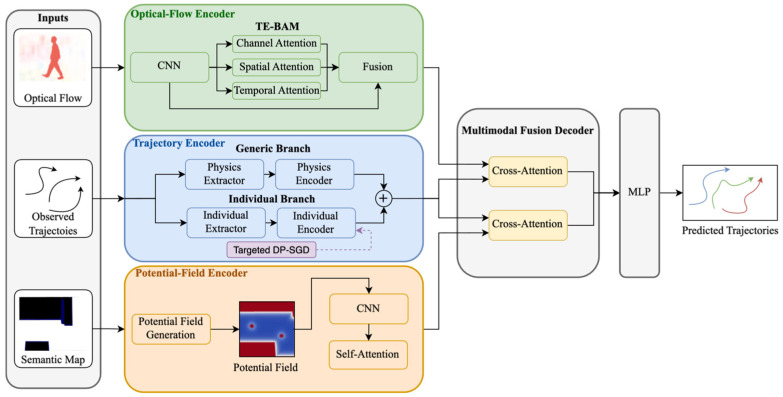
The framework of the CAMP. Black arrows denote the main information flow among modules. The purple dashed arrow indicates the targeted DP-SGD operation applied to the individual branch during private fine-tuning. The colored curves in the output box are schematic illustrations of predicted future trajectories.

Let $p_{i}^{t}\in R^{2}$ denote the 2D position of pedestrian i at time t. The observed history and prediction horizon are $X_{i}=\{p_{i}^{1},\ldots ,p_{i}^{T_{obs}}\}\in R^{T_{obs}\times 2},Y_{i}=\{p_{i}^{T_{obs}+1},\ldots ,p_{i}^{T_{obs}+T_{pred}}\}\in R^{T_{pred}\times 2}$, where $T_{obs}=8$ and $T_{pred}=12$. As shown in Figure 1, CAMP has three encoders and one decoder: (i) a trajectory encoder that learns motion patterns while embedding targeted differential privacy to protect individual behaviors; (ii) an optical-flow encoder with TE-BAM attention module that captures motion-centric scene dynamics; (iii) a potential-field encoder that models social and environmental interactions via physics-inspired potential fields; and (iv) a multimodal fusion decoder that outputs probabilistic future trajectory distributions.

### 4.2. Trajectory Encoder and Targeted Private Fine-Tuning

Historical trajectories contain both shared motion regularities and individualized behavioral tendencies. The former reflect population-level kinematic and interaction priors, while the latter may encode sensitive personalized habits. To distinguish these two factors, the trajectory encoder in CAMP is designed with two parallel branches: a generic branch and an individual branch. Given the observed trajectory $X_{i}$, the two branches first extract complementary feature representations through separate feature extractors.

Generic Extractor. The generic branch aims to capture physically plausible and population-level motion regularities. It encodes trajectory characteristics related to displacement continuity, velocity consistency, path efficiency, and motion predictability, producing a generic feature vector $f_{i}^{gen}\in R^{8\times 9}$.

Individual Extractor. The individual branch focuses on personalized motion tendencies. It encodes fine-grained kinematic descriptors, including velocity statistics, acceleration variation, heading change, curvature, and trajectory smoothness, producing an individual feature vector $f_{i}^{ind}\in R^{8\times 11}$.

The two feature vectors are then processed by separate temporal encoders to capture long-range dependencies, yielding latent representations $z_{i}^{ind}$ and $z_{i}^{gen}\in R^{256}$. Here, the pathway formed by the individual extractor and its temporal encoder is referred to as the individual branch, while the corresponding pathway for $f_{i}^{gen}$ is referred to as the generic branch. The two latent vectors are concatenated and projected through a feed-forward layer to obtain the final trajectory embedding $f_{traj}\in R^{256}$. This embedding is then passed to the multimodal fusion decoder.

Our privacy mechanism is implemented via a two-stage training process. In the first stage, the full model is pretrained on public data to learn reusable scene-level and population-level motion knowledge. In the second stage, all parameters are frozen except those of the individual branch, which is then fine-tuned on private data using differentially private stochastic gradient descent (DP-SGD). The motivation for this design is that privacy protection is mainly associated with individualized motion tendencies, whereas the generic branch and the context encoders primarily capture shared knowledge that should be preserved as much as possible. Therefore, DP noise is introduced only in the private adaptation of the individual branch, rather than throughout the entire model-training process.

Let $\theta^{ind}$ denote the trainable parameters of the individual branch during private fine-tuning. For a mini-batch B, the per-sample gradient for sample i∈B is first computed as follows:

(5) $$g_{i}=\nabla_{\theta^{ind}}l(\theta^{ind};x_{i})$$

Each gradient is then clipped to an $l_{2}$-norm bound C:

(6) $${clip}_{C}(g_{i})=g_{i}\cdot min\left(1,\frac{C}{∥g_{i}{∥}_{2}}\right)$$

The clipped gradients are aggregated and perturbed with Gaussian noise:

(7) $$\tilde{g}=\frac{1}{|B|}\left(\sum_{i\in B}{clip}_{C}(g_{i})+N(0,\sigma^{2}C^{2}I)\right)$$

where C is the clipping norm and σ is the noise multiplier. The privacy budget $\left(\varepsilon ,\delta \right)$ is determined by the clipping bound, noise level, sampling rate, and the number of update steps under DP accounting. This targeted private fine-tuning strategy is intended to limit the influence of each private trajectory on the individualized branch while retaining the shared motion priors and scene-level knowledge already captured by the generic branch and the other frozen modules.

### 4.3. Optical-Flow Encoder with TE-BAM

To complement trajectory-based motion intent with dynamic scene cues, CAMP employs an optical-flow encoder enhanced by a Temporally Enhanced Bottleneck Attention Module (TE-BAM). This branch operates on motion-centric optical-flow representations aligned with the observation window. Its role is to capture transient scene dynamics, such as pedestrian motion, obstacle movement, and background flow variation, while reducing direct dependence on explicit appearance information. The TE-BAM module further refines flow features through temporal, channel, and spatial attention, enabling the encoder to emphasize salient motion patterns while maintaining temporal consistency.

Given an optical-flow sequence aligned with the observed trajectory window $\{O_{t}{\}}_{t=1}^{T_{\mathrm{obs}}}$, each frame is firstly processed by a convolutional stem to produce frame-level flow features $F_{t}=\phi \left(O_{t}\right)\in R^{64\times H\times W}$, where ϕ(⋅) denotes the feature extractor of the optical-flow branch. The frame-level features are then stacked into a spatio-temporal tensor $F=\left[F_{1},F_{2},\ldots ,F_{T_{\mathrm{obs}}}\right]\in R^{T_{\mathrm{obs}}\times 64\times H\times W}$. This tensor is then fed into the TE-BAM module, which applies three parallel attention branches along the channel, spatial, and temporal dimensions. The three attention outputs are then fused through a bottleneck projection to obtain the enhanced flow representation $\tilde{F}$.

Temporal Attention. To capture frame-to-frame dependencies and suppress transient noise, TE-BAM introduces a lightweight temporal self-attention mechanism. For each frame feature $F_{t}$, spatial global average pooling is first applied to obtain a compact descriptor:

(8) $$p_{t}={GAP}_{spatial}(F_{t})\in R^{C_{flow}}$$

This descriptor is projected into a temporal embedding space to form query and key vectors:

(9) $$q_{t}=W_{q}p_{t},k_{t}=W_{k}p_{t}$$

where $q_{t},k_{t}\in R^{d_{k}}$. The temporal correlation between frame t and frame u is then computed by scaled dot-product attention:

(10) $$\alpha_{t,u}=\frac{\exp\left(q_{t}^{⊤}k_{u}/\sqrt{d_{k}}\right)}{\sum_{v=1}^{T_{obs}}exp\left(q_{t}^{⊤}k_{v}/\sqrt{d_{k}}\right)}$$

Here, $\alpha_{t,u}$ measures the relevance of frame u with respect to frame t.

Based on the temporal attention matrix α, we further compute a frame-level importance score by aggregating the total attention received by each frame:

(11) $$w_{t}=\frac{\exp\left(\sum_{u=1}^{T_{obs}}\alpha_{u,t}\right)}{\sum_{\tau =1}^{T_{obs}}exp\left(\sum_{u=1}^{T_{obs}}\alpha_{u,\tau }\right)}$$

Thus, $w_{t}\in (0,1)$ and $\sum_{t=1}^{T_{obs}}w_{t}=1$. These weights quantify global temporal saliency and are reshaped into a temporal attention map:

(12) $$A_{t}\in R^{T_{obs}\times 1\times 1\times 1}$$

which is broadcast and multiplied with F.

Channel Attention. The channel attention branch adaptively reweights feature channels to highlight dimensions that provide informative motion cues. For each frame $F_{t}$, spatial global average pooling generates a per-frame descriptor:

(13) $$u_{t}={GAP}_{spatial}\left(F_{t}\right)$$

This descriptor is passed through a two-layer excitation network to get the per-frame channel weight:

(14) $$a_{c,t}=\sigma (MLP(u_{t}))$$

where σ(⋅) denotes the sigmoid activation. The per-frame channel weights are further aggregated into a temporally weighted channel attention map using the temporal attention weights $w_{t}$ calculated by the temporal attention module described above:

(15) $$A_{c}=\sum_{t=1}^{T_{\mathrm{obs}}}w_{t}a_{c,t}$$

where $w_{t}\in \left[0,1\right]$ denotes the normalized frame-level importance derived from the temporal attention matrix α, is the frame-level importance score computed in the temporal attention module. $A_{c}$ is broadcast to match the dimensions of F. This design allows globally important frames to contribute more strongly to channel reweighting.

Spatial Attention. The spatial branch identifies regions with salient motion patterns in the scene by aggregating information along the channel dimension and generating a 2D spatial attention map. Average-pooling and max-pooling are applied along the channel axis and concatenated, followed by a lightweight convolution:

(16) $$A_{s}=Conv\left(\left[{AvgPool}_{C}\left(F\right);{MaxPool}_{C}\left(F\right)\right]\right)$$

This spatial mask enables the encoder to focus on dynamic areas, such as pedestrian clusters or moving obstacles, while reducing emphasis on static regions, such as the ground plane or buildings.

The three attention maps are fused within a bottleneck projection to form the enhanced flow representation:

(17) $$\tilde{F}=W_{b}\left(A_{c}⊙F+A_{s}⊙F+A_{t}⊙F\right)$$

where ⊙ denotes scaling applied to each element, and $W_{b}$ is a fusion projection layer. The resulting feature tensor $\tilde{F}$ is globally pooled and projected to obtain the optical-flow embedding $f^{flow}\in R^{256}$. This embedding provides motion-centric dynamic scene cues that complement the trajectory and potential-field representations in the subsequent multimodal decoder.

### 4.4. Potential-Field Encoder with Physics-Guided Modeling

**Figure 2:**
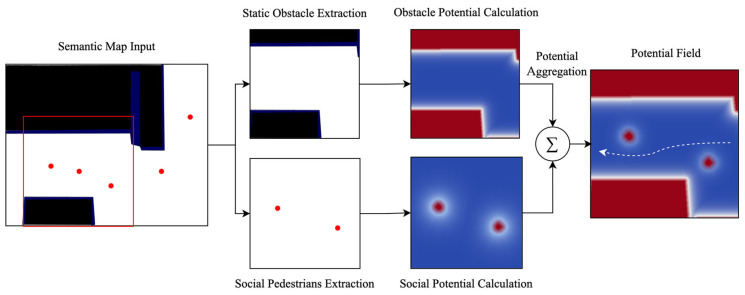
Illustration of the potential field construction process. The red box denotes the local ego-centric grid centered on the target pedestrian, red dots indicate pedestrians or obstacle-related sources used for potential computation, and arrows illustrate the construction flow from semantic and social cues to the final local potential field.

Figure 2 illustrates the construction of the local potential field used by CAMP. For each target pedestrian at each observed time step, we first define an ego-centric K × K grid centered at the pedestrian’s current position. Based on the semantic map, obstacle regions are identified and converted into obstacle sources in the local coordinate system. At the same time, neighboring pedestrians provide dynamic social interaction sources through their current positions and relative motion states. The obstacle potential and the social potential are then computed on each grid cell and summed to form the final local energy map. By repeating this process over the observation window, we obtain a spatio-temporal stack of local potential fields, which is further encoded by a lightweight convolutional network to produce the potential-field representation used in multimodal fusion.

For each pedestrian i at time step t, we construct a local potential field $U_{i}^{t}\in R^{K\times K}$ centered at the current position $p_{i}^{t}$. The field is discretized into a 32 × 32 grid, i.e., K = 32, with a grid resolution of Δ = 0.25 m per cell. Let m,n∈{1,…,K} index the cells in this field, and let Δ denote the grid resolution. We define the field center index as $m_{0}=n_{0}=\lfloor K/2\rfloor +1$. The world coordinate of the cell center (m,n) is as follows:

(18) $$c_{m,n}^{i,t}=p_{i}^{t}+[(m-m_{0})\Delta ,(n-n_{0})\Delta {]}^{⊤}$$

Each cell is assigned a scalar potential value $U_{i}^{t}(m,n)$ that reflects the traversal cost of the corresponding local region. The total potential is defined as the sum of the obstacle potential and the social potential:

(19) $$U_{i}^{t}(m,n)=U_{i,t}^{obs}(m,n)+U_{i,t}^{soc}(m,n)$$

Obstacle potential. From the semantic map, we extract centers of obstacle cells and denote them as a set $O=\{o_{k}\}$. The obstacle potential on the field is computed by explicitly aggregating repulsive contributions from all obstacle sources:

(20) $$U_{i,t}^{obs}(m,n)=\sum_{o_{k}\in O}\alpha_{o}exp(-\beta_{o}∥c_{m,n}^{i,t}-o_{k}{∥}^{2})$$

where $\alpha_{o},\beta_{o}>0$ are learnable parameters controlling the magnitude and spatial decay of obstacle influence. This formulation assigns higher potential to locations closer to obstacles while keeping free regions at low cost, producing a smooth and interpretable local energy landscape around pedestrian i.

Social potential. Let $N_{i}^{t}$ denote the set of neighboring pedestrians around pedestrian i at time t, with positions $p_{j}^{t}$ and velocities $v_{j}^{t}$. The social interaction potential is computed as follows:

(21) $$U_{i,t}^{soc}(m,n)=\sum_{j\in N_{i}^{t}}\alpha_{s}exp(-\beta_{s}∥c_{m,n}^{i,t}-p_{j}^{t}{∥}^{2})\;exp(-\gamma_{s}\Delta v_{i,j}^{t}),$$

where $\Delta v_{i,j}^{t}=∥v_{i}^{t}-v_{j}^{t}∥$, and $\alpha_{s},\beta_{s},\gamma_{s}>0$ are learnable coefficients controlling interaction strength, spatial decay, and velocity sensitivity. The first exponential term captures distance-based interpersonal influence, while the second term modulates this influence according to relative motion difference. As a result, regions with higher collision risk or stronger interaction pressure receive larger social potential values.

The resulting field $U_{i}^{t}$ provides an ego-centric energy map that explicitly describes the local spatial cost around pedestrian i at time t. By stacking the fields over the observation window, we obtain $U_{i}\in R^{T_{obs}\times K\times K}$, which is then processed by a lightweight convolutional encoder to extract hierarchical spatial features and generate the potential-field embedding $f^{pot}=\psi_{pot}(U_{i})\in R^{256}$. This embedding is subsequently fused with the trajectory embedding $f^{traj}$ and the optical-flow embedding $f^{flow}$ in the multimodal decoder. Through this design, the potential-field branch provides an explicit and interpretable spatial prior for socially compliant prediction.

### 4.5. Multimodal Fusion Decoder

To generate socially compliant predictions that remain sensitive to scene dynamics and spatial constraints, CAMP employs a multimodal decoder with two parallel cross-attention modules. This decoder integrates trajectory intent, optical-flow-based motion cues, and potential-field-based interaction constraints into a unified representation for probabilistic forecasting.

Before fusion, the trajectory embedding $f^{traj}$, the optical-flow embedding $f^{flow}$, and the potential-field embedding $f^{pot}$ are first normalized and projected into a shared latent space to ensure dimensional consistency across modalities. Let the projected representations be denoted by the same symbols for simplicity.

The decoder contains two cross-attention branches. In both branches, the trajectory embedding serves as the query, while the auxiliary modality provides the key–value pair. Specifically, the flow-guided branch is defined as follows:

(22) $$h^{flow}=Attn\left(f^{traj},f^{flow},f^{flow}\right)$$

and the potential-guided branch is defined as follows:

(23) $$h^{pot}=Attn\left(f^{traj},f^{pot},f^{pot}\right)$$

where Attn(Q,K,V) denotes a multi-head cross-attention operator with four attention heads, corresponding to a per-head dimension of 64.

This dual-branch design allows the decoder to incorporate two complementary sources of context. The optical-flow branch emphasizes dynamic scene motion cues, while the potential-field branch provides explicit spatial interaction constraints. Their outputs are concatenated and refined through a lightweight fusion layer to obtain the final multimodal representation:

(24) $$h=\phi_{fuse}([h^{flow};h^{pot}])$$

where $\left[\cdot ;\cdot \right]$ denotes vector concatenation and $\phi_{fuse}(\cdot )$ is a learnable fusion function.

The fused representation h is then fed into an MLP to predict the parameters of a bivariate Gaussian distribution at each future time step. Specifically, the decoder outputs the mean vector and covariance parameters of the predictive distribution:

(25) $$(\mu_{t},\Sigma_{t})=\psi_{out}(h_{t}),t=1,\ldots ,T_{pred}$$

where $\mu_{t}\in R^{2}$ denotes the predicted position mean and $\Sigma_{t}$ denotes the corresponding covariance matrix. This formulation enables the decoder to model predictive uncertainty and to generate probabilistic future trajectories.

During training, the decoder is optimized using the negative log-likelihood loss over the ground-truth future trajectory under the predicted Gaussian distributions:

(26) $$L_{NLL}=-\sum_{t=1}^{T_{pred}}logp\left(y_{t}|\mu_{t},\Sigma_{t}\right)$$

Through this design, the decoder integrates motion intent, dynamic scene information, and explicit spatial constraints within a unified prediction process.

## 5. Experiments

This section assesses the proposed CAMP framework through comprehensive experiments on various benchmark datasets. We first describe the experimental settings, including datasets, metrics, baselines, and implementation details. We then present a comparative performance analysis combining quantitative metrics and qualitative visualizations. Finally, we present an ablation study to assess the contribution of each component module and analyze the privacy-utility trade-off.

### 5.1. Experimental Settings

Datasets. We perform experiments on the widely used ETH/UCY benchmark, which includes five real-world crowd scenes: ETH, HOTEL, UNIV, ZARA1, and ZARA2. These datasets consist of pedestrian trajectories collected in outdoor campus and urban environments, each with unique spatial layouts and motion interaction patterns. Each scene provides annotated 2D coordinates of pedestrians over time, recorded at 2.5 Hz. Following standard practice, we observe 8 time steps (3.2 s) and predict the next 12 time steps (4.8 s). The trajectories are smoothed and converted into local coordinates centered on each pedestrian’s last observed position to ensure comparability across scenes. To further assess generalization, we adopt the standard leave-one-out protocol: one scene is used for testing while the others are used for training. This method ensures cross-scene robustness and allows for a fair comparison with previous studies.

Two-Stage Training Protocol. For each leave-one-out fold, the four non-test scenes form the source training pool. Since ETH/UCY does not natively provide a public/private split, we further partition this source pool into a public subset $D_{pub}$ and a private subset $D_{priv}$ to simulate a privacy-constrained adaptation setting. In Stage I, the full CAMP model is pretrained on $D_{pub}$ using standard optimization to learn reusable scene-level and population-level motion priors. In Stage II, all modules are frozen except for the individual branch of the trajectory encoder, which is fine-tuned on $D_{priv}$ using DP-SGD. The held-out test scene is never used in either training stage. Under this protocol, the formal privacy guarantee discussed in this work is defined with respect to $D_{priv}$, rather than the entire two-stage training pipeline. This protocol is designed to reflect the distinction between shared motion knowledge and individualized motion tendencies: Stage I learns reusable shared priors, whereas Stage II adapts only the branch intended to encode personalized motion patterns.

Evaluation Metrics. We use the two most common metrics for pedestrian trajectory prediction: Average Displacement Error (ADE), the mean Euclidean distance between predicted and ground-truth trajectories over all time steps; and Final Displacement Error (FDE), the Euclidean distance between predicted and ground-truth final positions. Because pedestrian motion is inherently multimodal, each model produces K samples for each input. We report $minADE_{K}$, and $minFDE_{K}$, representing the minimum ADE and FDE across the K generated trajectories. Throughout our experiments, K=20 unless otherwise stated.

Baseline Methods. To thoroughly evaluate CAMP, we compare it with representative pedestrian trajectory prediction methods from several methodological categories. Specifically, S-GAN [3] and SoPhie [37] are generative adversarial approaches, where S-GAN mainly models socially acceptable future trajectories from historical coordinates and SoPhie further incorporates physical scene context. S-STGCNN [4] and SGCN [38] are graph-based methods that capture pedestrian interactions through spatio-temporal graph structures. Transformer [39], STAR [5], SIF-TF [26], and STP [24] are Transformer-based forecasting models, where Transformer provides a basic self-attention framework for trajectory forecasting, STAR further models spatio-temporal interactions among multiple pedestrians, SIF-TF integrates pedestrian intention with semantic scene information, and STP improves trajectory prediction by exploiting sparse interaction structures within a Transformer-style encoder-decoder framework. MTN [27] is a multimodal Transformer-based method that introduces optical-flow-based motion representations to capture transient scene dynamics through multi-level fusion. LED [23] and IDP [22] are diffusion-based stochastic prediction models, where LED improves trajectory generation efficiency through a trainable leapfrog initializer, and IDP enhances multimodal future prediction by modeling diverse trajectory distributions with a diffusion-based generation process.

Implementation Details. We report ${minADE}_{K}$ and ${minFDE}_{K}$ with K=20 samples per input. Unless otherwise stated, all encoders and the multimodal decoder use a hidden dimension of 256 with four attention heads. All MLPs use ReLU activation and a dropout rate of 0.1. The potential-field map is discretized into a 32×32 grid. CAMP is trained with Adam using an initial learning rate of $1\times 10^{-3}$, which gradually decayed during training, a batch size of 128, and 500 epochs on a single NVIDIA RTX 3090 GPU (24 GB). All reported results are averaged over five random seeds. During inference, we draw K=20 independent rollouts for each input and compute the minimum ADE and FDE over the generated samples. Static scene semantics are precomputed using DeepLabv3+ [40] from MMSegmentation v1.2.2, and dense optical flow is precomputed using RAFT [41] from torchvision v0.23.0. The resulting semantic maps and optical-flow fields are aligned with the eight-step observation window and then used as inputs to the potential-field encoder and the TE-BAM-based optical-flow encoder, respectively. During the private fine-tuning stage, only the individual branch of the trajectory encoder is updated, while all remaining modules remain frozen. We apply DP-SGD with a per-sample gradient clipping norm of C=1.0 and fix $\delta =10^{-5}$. For each target privacy budget ε∈{10,8,5}, the corresponding noise multiplier σ is selected using a Rényi differential privacy accountant with sampling rate $q=\frac{\mathrm{batch}}{N}$ and total update steps $T=\mathrm{epochs}\times \frac{N}{\mathrm{batch}}$, where N denotes the size of the private fine-tuning subset in each leave-one-out fold. Under this protocol, the released model satisfies $\left(\varepsilon ,\delta \right)$-DP with respect to the private fine-tuning data used to adapt the individual branch, rather than the entire end-to-end training pipeline. The corresponding σ values and privacy-utility results are reported in the privacy-utility table.

### 5.2. Comparative Performance Analysis

In this section, we evaluate CAMP from both quantitative and qualitative perspectives on the ETH/UCY benchmark. Since the compared methods differ in input modalities and training protocols, the results in Table 1 are interpreted as empirical comparisons under the reported settings. A more explicit comparison of input modalities is provided in Table 1.

Quantitative Results. Table 2 reports the quantitative results on the ETH/UCY benchmark. CAMP achieves competitive overall performance, with an average ADE/FDE of 0.20/0.36. Compared with earlier adversarial, graph-based, and basic attention-based baselines, CAMP yields substantially lower prediction errors across most scenes. Relative to recent strong methods, CAMP matches the best reported average ADE and remains close to the strongest average FDE results. At the scene level, CAMP obtains the lowest ADE on ETH (0.35) and matches the best reported result on UNIV (0.23/0.44), while remaining close to the strongest methods on HOTEL, ZARA1, and ZARA2. These results suggest that integrating trajectory history, optical-flow cues, and potential-field constraints provides complementary information for pedestrian trajectory prediction. Under the reported private fine-tuning setting with ε = 10, DP-CAMP achieves 0.22/0.39, corresponding to absolute increases of 0.02 in ADE and 0.03 in FDE relative to the non-private CAMP model. This indicates that the targeted privacy mechanism preserves a substantial part of the predictive utility under the studied privacy budget. A more detailed privacy-utility analysis under different ε values is provided in Section 5.4.

**Figure 3:**
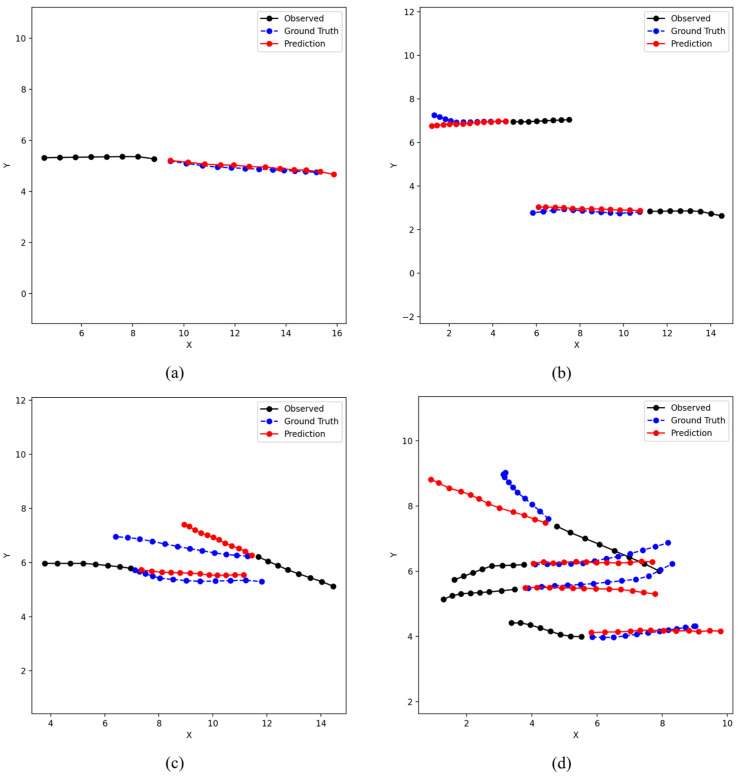
Qualitative comparison of predicted trajectories in representative interaction scenarios on the ETH/UCY benchmark. (**a**) Single walking; (**b**) same-direction walking; (**c**) opposite-direction walking; (**d**) multi-person mixed walking. Black lines denote observed trajectories, blue lines denote ground-truth futures, and red lines denote predicted futures.

Qualitative Analysis. To further examine CAMP’s predictive behavior, Figure 3 presents representative forecasts in four typical scenarios: (a) single walking; (b) same-direction walking; (c) opposite-direction walking; and (d) multi-person mixed walking.

**Figure 4:**
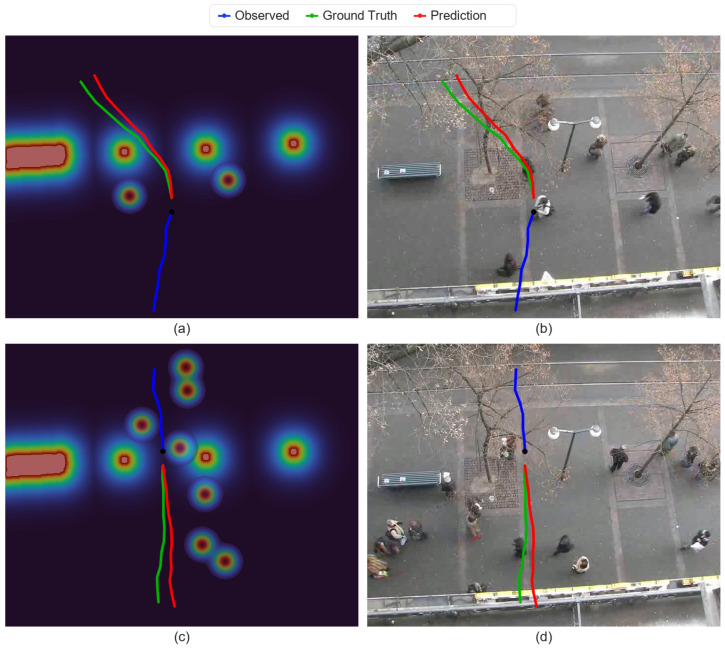
Representative potential-field heatmaps and their corresponding trajectory predictions on the ETH/UCY benchmark. (**a**) Potential-field heatmap of the first example, where high-potential regions are formed around obstacles and nearby interaction sources; (**b**) scene-level trajectory prediction corresponding to (**a**); (**c**) potential-field heatmap of the second example, where multiple high-potential regions indicate denser local interaction constraints; (**d**) scene-level trajectory prediction corresponding to (**c**). Blue, green, and red curves denote the observed trajectory, ground-truth future trajectory, and predicted future trajectory, respectively. Warmer colors in the heatmaps indicate higher potential values (i.e., higher local traversal cost), whereas darker regions indicate lower potential values and relatively freer space.

In the single-walking case, the predicted trajectory remains smooth and follows the overall motion trend of the ground truth. In the same-direction case, the predicted trajectories maintain relatively consistent spacing and direction among neighboring pedestrians. In the opposite-direction case, the predicted paths exhibit an avoidance tendency near potential conflict regions, which is qualitatively consistent with the role of the potential-field branch. In the multi-person mixed case, the predictions remain coherent under more complex interactions, with different pedestrians showing distinct local responses such as maintaining direction, slight turning, or speed adjustment. Overall, these examples suggest that CAMP can combine motion history, dynamic scene cues, and interaction constraints in diverse crowd scenarios. To further support the interpretability claim, we additionally visualize representative potential-field heatmaps and their corresponding predicted trajectories in Figure 4.

Figure 4 further illustrates representative potential-field heatmaps together with the corresponding trajectory predictions. In the heatmaps, warmer colors indicate regions with higher traversal cost induced by nearby pedestrians and obstacle sources, while darker regions correspond to lower-cost free space. In the first example, the predicted future bends away from a high-potential region and remains closer to the low-cost corridor, which is consistent with the avoidance behavior expected in the scene. In the second example, the potential distribution is more concentrated around neighboring pedestrians, and the predicted trajectory follows a relatively straight, low-cost path with only limited lateral deviation. These cases provide qualitative evidence that the potential-field branch supplies an explicit and visually interpretable spatial prior, rather than serving only as an implicit auxiliary feature.

### 5.3. Ablation Study

To verify the contribution of each component, we conduct systematic ablation experiments on the ETH/UCY benchmark in Table 3. We first examine the effect of the main modality branches, including the trajectory encoder alone (T), the trajectory encoder with the optical-flow branch (T+O), and the trajectory encoder with the potential-field branch (T+P). We then further evaluate three variants of the full multimodal model by replacing the attention module in the optical-flow encoder with standard attention (SA), bottleneck attention module (BAM), and the proposed temporally enhanced bottleneck attention module (TE-BAM), respectively. Finally, we include a differentially private variant of the full model, denoted as DP-T+O+P (TE-BAM), to assess the utility impact of the targeted privacy mechanism in the same multimodal setting.

As shown in Table 3, both additional branches improve performance over the trajectory-only baseline. Compared with T, T+O reduces ADE/FDE from 0.54/1.17 to 0.41/0.89, indicating that optical-flow cues provide useful dynamic context. T+P further improves the result to 0.33/0.54, suggesting that explicit spatial interaction constraints are particularly beneficial, especially for reducing long-horizon prediction error.

When all three branches are combined, the attention design in the optical-flow encoder further affects performance. T+O+P (SA) achieves 0.25/0.43, and replacing SA with BAM improves the result to 0.22/0.38. The full model with TE-BAM achieves the best performance, with 0.20/0.36, indicating that the proposed temporal enhancement provides additional benefit beyond standard attention and BAM.

The differentially private variant, DP-T+O+P (TE-BAM), achieves 0.22/0.39, corresponding to absolute increases of 0.02 in ADE and 0.03 in FDE relative to the non-private model. This suggests that the targeted privacy mechanism introduces a moderate utility loss while preserving the overall advantage of the full multimodal design. A more detailed privacy-utility analysis is provided in Section 5.4.

These results suggest that the gains of CAMP do not arise simply from adding more inputs, but from assigning different modeling roles to them within a unified architecture: the optical-flow branch mainly contributes motion-centric transient context, whereas the potential-field branch contributes explicit spatial interaction constraints.

### 5.4. Privacy Analysis and Utility Trade-Off

Threat Model and Scope. In this work, the formal privacy guarantee of CAMP is defined with respect to the private fine-tuning stage of the individual branch. Specifically, the protected data are the private trajectories used during this adaptation stage, while the model is first pretrained on public data to learn reusable scene-level and population-level motion priors. We consider a black-box adversary who can query the released model and observe its prediction outputs, but does not directly access the private fine-tuning trajectories. Under this setting, the privacy concern is whether individual-specific motion patterns from the private fine-tuning data may be memorized and exposed through the adapted model. Accordingly, the DP guarantee in this work applies to the dependence of the individual branch on the private fine-tuning dataset, rather than to the entire end-to-end training pipeline. In particular, freezing the generic branch and the remaining modules during private fine-tuning prevents them from absorbing additional information from the private adaptation data, but does not retroactively remove any information that may have been learned during public pretraining.

Theoretical Privacy Guarantee. The privacy guarantee of CAMP follows from the two-stage training protocol and the post-processing property of differential privacy. During public pretraining, the full model is trained on public data only, so this stage does not consume any privacy budget. During private fine-tuning, all modules are frozen except for the individual branch of the trajectory encoder, which is updated using DP-SGD. Therefore, the only trainable parameters that directly depend on the private fine-tuning data are those of the individual branch. Since all subsequent computations in the frozen generic branch, the optical-flow encoder, the potential-field encoder, the multimodal fusion decoder, and the output layer operate on the resulting private representation without further accessing the private dataset, they can be regarded as post-processing of a differentially private intermediate output. Under the stated threat model, the released model therefore satisfies (ε,δ)-DP with respect to the private fine-tuning data used to adapt the individual branch. The privacy cost is determined solely by the DP-SGD updates applied to this branch and is accounted for using Rényi differential privacy (RDP).

Quantitative Privacy-Utility Trade-off. Under differential privacy, smaller values of ε correspond to stronger privacy protection but usually lower utility, whereas larger values correspond to weaker privacy and typically better utility. However, the practical interpretation of ε depends on the threat model, the unit of privacy, the value of δ, and the deployment context, rather than on a universally fixed threshold [36]. In this work, the reported values ε∈{10, 8, 5} are therefore interpreted as operating points for analyzing the utility–privacy trade-off under the private fine-tuning threat model defined in Section 3.2, rather than as universal deployment-level guarantees. Among them, ε=10 corresponds to the loosest reported privacy setting, ε=8 is stricter, and ε=5 is stricter still.

This interpretation is also consistent with the broader DP-SGD literature. Classical work, such as Abadi et al., characterized single-digit ε as a modest privacy regime in deep learning [36], while more recent differentially private vision studies often report results across a range such as ε∈[1, 8] to characterize practical privacy-utility trade-offs [30]. At the same time, direct comparison with trajectory privacy studies should be made cautiously, because many of them focus on private trajectory release or local privacy mechanisms rather than differentially private model fine-tuning. Therefore, the main purpose of Table 4 is not to claim that ε=10 constitutes strong privacy in every real-world deployment, but to show how utility changes across several formally defined DP operating points within our specific training protocol.

Table 4 reports the corresponding utility degradation under these privacy settings. Moving from the non-private setting to ε=10 increases ADE/FDE from 0.20/0.36 to 0.22/0.39. Tightening the privacy budget further to ε=8 yields 0.23/0.41, and ε=5 yields 0.25/0.46. Overall, the prediction performance degrades gradually as privacy becomes stricter, which is consistent with the expected utility loss introduced by DP noise. Accordingly, we do not claim that ε=10 provides strong privacy for all deployment scenarios. Rather, under the threat model considered in this work, ε=10 should be viewed as the loosest reported operating point that still provides a formal (ε, δ)-DP guarantee for the private fine-tuning data, whereas ε=5 represents a stricter but less utility-preserving setting. In applications with stronger privacy requirements, smaller ε values and additional empirical privacy auditing may be preferred.

### 5.5. Efficiency and Complexity Analysis

To assess the deployment overhead of CAMP, we compare its inference efficiency with controlled baselines under the same codebase and benchmark protocol. As shown in Table 5, CAMP has 91.7 MB peak GPU memory and an inference latency of 3.74 ms per prediction window, corresponding to 267.2 FPS. When CPU-side potential-field construction is included, the end-to-end latency under the 10-neighbor setting becomes 4.98 ms, corresponding to 200.7 FPS. Compared with the Transformer and Social-LSTM baselines, CAMP is not the lightest model, but the measured overhead remains moderate under the reported ETH/UCY setting. The results also indicate that the main additional cost comes from local potential-field construction rather than from the multimodal predictor alone.

To further examine scalability, Table 6 reports the runtime across different potential-field grid sizes. The GPU inference latency remains nearly unchanged from 32 × 32 to 96 × 96 grids, indicating that the main network forward cost is relatively stable. In contrast, the CPU-side field builder grows substantially with both grid resolution and neighborhood size. For example, under 40 neighbors, the builder latency increases from 4.00 ms at 32 × 32 to 21.91 ms at 64 × 64 and 48.14 ms at 96 × 96, while the corresponding end-to-end latency rises from 7.14 ms to 25.10 ms and 51.29 ms. Temporary builder memory shows a similar trend, increasing from 0.664 MB to 5.977 MB. Under fixed $T_{obs}$ and a K×K grid, the dominant cost of potential-field construction scales approximately as $O(T_{obs}K^{2}(|O_{local}|+|N|))$, where ∣N∣ denotes the number of neighbors and $|O_{local}|$ denotes the number of local obstacle sources. These results indicate that the main scalability bottleneck of CAMP lies in local potential-field construction, which supports our use of a 32 × 32 grid as a practical trade-off between efficiency and modeling granularity.

## 6. Conclusions

This paper presented CAMP, a context-aware and privacy-aware framework for pedestrian trajectory prediction built around a role-aligned multimodal architecture. In CAMP, the trajectory encoder separates shared motion regularities from individualized motion tendencies, the optical-flow branch captures motion-centric transient scene dynamics, and the potential-field branch provides an explicit spatial cost prior for socially compliant forecasting. Based on this design, targeted DP-SGD is applied only to the individual branch during private fine-tuning under the stated threat model.

Experimental results on the ETH/UCY benchmark indicate that these complementary components improve trajectory prediction under the reported setting. In particular, the optical-flow branch contributes dynamic scene cues, while the potential-field branch introduces explicit interaction constraints that are qualitatively consistent with avoidance-aware prediction behavior. In addition, the private variant of CAMP maintains a reasonable utility-privacy trade-off across the reported privacy budgets, suggesting that restricting private adaptation to the individualized branch helps preserve much of the shared motion and scene knowledge learned during public pretraining.

Several limitations should also be noted. First, the interpretation of the privacy budget ε is context-dependent, and the reported values should be understood as operating points in the utility-privacy analysis under the studied training protocol rather than as universal deployment-level guarantees. Second, optical flow is not equivalent to strict anonymization; in this work, it is used as a motion-centric representation that reduces direct reliance on raw RGB appearance cues. Third, the construction of local potential fields introduces additional computational overhead, which may affect scalability in denser scenes or larger-scale deployments. Fourth, the current decoder models predictive uncertainty with a Gaussian parameterization and does not explicitly represent mixture-style multimodal futures.

Future work will therefore proceed in three directions. First, we will explore adaptive privacy budgeting strategies under different scene complexities and data regimes. Second, we will investigate more robust appearance-reduced sensing or representation forms, such as LiDAR or related motion-centric modalities, to improve performance under challenging visual conditions. Third, we will reduce the computational overhead of potential-field construction and study more expressive forecasting decoders for modeling diverse future motion patterns across more heterogeneous datasets.

## Figures and Tables

**Table 1:** Input modality comparison of CAMP and representative baselines.

Methods	Trajectory	Social Context	Scene Context	Optical Flow
S-GAN	✓	✓		
SoPhie	✓	✓	✓	
S-STGCNN	✓	✓		
SGCN	✓	✓		
Transformer	✓			
STAR	✓	✓		
SIF-TF	✓	✓	✓	
MTN	✓			✓
LED	✓	✓		
IDP	✓	✓		
STP	✓	✓		
CAMP	✓	✓	✓	✓
DP-CAMP	✓	✓	✓	✓

Note: A check mark (✓) indicates that the corresponding input modality or contextual information is used by the method.

**Table 2:** Quantitative comparison on the ETH/UCY dataset in terms of ADE/FDE (lower is better).

Methods	ETH	HOTEL	UNIV	ZARA1	ZARA2	AVG
S-GAN	0.87/1.62	0.67/1.37	0.76/1.52	0.35/0.68	0.42/0.84	0.61/1.21
SoPhie	0.70/1.43	0.76/1.67	0.54/1.24	0.30/0.63	0.38/0.78	0.51/1.15
S-STGCNN	0.64/1.11	0.49/0.85	0.44/0.79	0.34/0.53	0.30/0.48	0.44/0.75
SGCN	0.63/1.03	0.32/0.55	0.37/0.70	0.29/0.53	0.25/0.45	0.37/0.65
Transformer	1.03/2.10	0.36/0.71	0.53/1.32	0.44/1.00	0.34/0.76	0.54/1.17
STAR	0.36/0.64	0.17/0.36	0.31/0.62	0.29/0.52	0.22/0.46	0.26/0.53
SIF-TF	0.29/0.55	0.12/0.23	0.32/0.70	0.23/0.23	0.20/0.44	0.23/0.47
MTN	0.58/0.86	0.19/0.57	0.32/0.43	0.24/0.37	0.15/0.21	0.30/0.49
LED	0.39/0.58	0.11/0.17	0.26/0.43	0.18/0.26	0.13/0.22	0.21/0.33
IDP	0.37/0.61	0.12/0.20	0.23/0.44	0.20/0.36	0.15/0.29	0.21/0.38
STP	0.36/0.51	0.11/0.16	0.24/0.41	0.17/0.31	0.13/0.23	0.20/0.32
CAMP	0.35/0.57	0.12/0.20	0.23/0.44	0.18/0.35	0.14/0.24	0.20/0.36
DP-CAMP	0.37/0.62	0.12/0.21	0.25/0.48	0.19/0.37	0.15/0.26	0.22/0.39

**Table 3:** Ablation results on the ETH/UCY dataset in terms of average ADE/FDE (lower is better).

Model	ADE	FDE
T	0.54	1.17
T+O	0.41	0.89
T+P	0.33	0.54
T+O+P (SA)	0.25	0.43
T+O+P (BAM)	0.22	0.38
T+O+P (TE-BAM)	0.20	0.36
DP-T+O+P (TE-BAM)	0.22	0.39

**Table 4:** Privacy-utility trade-off under different privacy budgets on the ETH/UCY dataset in terms of ADE/FDE (lower is better).

ε	σ	ADE	FDE
∞ (No DP)	-	0.20	0.36
10.0	0.67	0.22	0.39
8.0	0.75	0.23	0.41
5.0	0.95	0.25	0.46

**Table 5:** Efficiency comparison of CAMP and representative baselines during inference. Inference latency measures GPU forward inference with preloaded inputs, while end-to-end latency additionally includes CPU-side potential-field construction for CAMP under the 10-neighbor setting.

Methods	Inference Latency (ms)	Inference FPS	End-to-End Latency @10N (ms)	End-to-End FPS @10N	Peak GPU Memory (MB)
Transformer	0.88	1137.6	0.88	1137.6	20.9
Social-LSTM	0.63	1586.6	0.63	1586.6	33.9
CAMP	3.74	267.2	4.98	200.7	91.7

**Table 6:** Scalability analysis of CAMP under different potential-field grid sizes. Inference latency measures GPU forward inference with preloaded inputs, while field construction time, end-to-end latency, and temporary builder memory additionally account for CPU-side potential-field construction under different neighborhood sizes.

Metric	32 × 32	64 × 64	96 × 96
Inference Latency (ms)	3.14	3.20	3.15
Inference FPS	318.6	312.6	317.8
Field Construction Time @10N (ms)	1.08	3.09	9.39
End-to-End Latency @10N (ms)	4.22	6.29	12.53
End-to-End FPS @10N	236.8	158.9	79.8
Field Construction Time @40N (ms)	4.00	21.91	48.14
End-to-End Latency @40N (ms)	7.14	25.10	51.29
End-to-End FPS @40N	140.1	39.8	19.5
Peak GPU Memory (MB)	91.7	91.7	91.9
Temporary Builder Memory @10N (MB)	0.195	0.781	1.758
Temporary Builder Memory @40N (MB)	0.664	2.656	5.977

## Data Availability

The original data presented in the study are openly available in ETH and UCY at https://github.com/crowdbotp/OpenTraj (accessed on 5 February 2026).

## Abbreviations

The following abbreviations are used in this manuscript:

CAMP	Context-Aware, Multimodal, and Privacy-Preserving (framework)
DP	Differential Privacy
DP-SGD	Differentially Private Stochastic Gradient Descent
DP-CAMP	DP version of CAMP
RDP	Rényi Differential Privacy
TE-BAM	Temporal-Enhanced Bottleneck Attention Module
ADE	Average Displacement Error
FDE	Final Displacement Error
SFM	Social Force Model
GNN	Graph Neural Network
GAN	Generative Adversarial Network
BEV	Bird’s-Eye View
FL	Federated Learning
MLP	Multi-Layer Perceptron
ReLU	Rectified Linear Unit
RGB	Red Green Blue (color channels)
LiDAR	Light Detection and Ranging
